# Malignant and Bony Tumors of the Pediatric Hand: A Review of Diagnosis and Treatment Strategies

**DOI:** 10.7759/cureus.86907

**Published:** 2025-06-28

**Authors:** Zuhair Zaidi, Daniel Villarreal Acha, Muaz Wahid, Amber McCranie, Sameer Sajjad, Jennifer Kargel

**Affiliations:** 1 Plastic Surgery, University of Texas Southwestern Medical School, Dallas, USA; 2 Surgery, Edward Via College of Osteopathic Medicine, Monroe, USA

**Keywords:** hand tumors, orthopedic hand surgery, pediatric hand surgery, plastic surgery, surgical oncology

## Abstract

Malignant or bony tumors of the pediatric hand, although uncommon, pose critical challenges due to their potential for local invasion, functional compromise, and, in rare cases, metastatic spread. This review explores a range of benign and malignant lesions, including osteochondromas, enchondromas, giant cell tumors, osteosarcoma, Ewing sarcoma, and melanoma, emphasizing their unique presentations in children compared to adults. The small anatomical space and high functional demands of the hand make accurate diagnosis and timely management essential to preserving growth potential and quality of life. By examining the clinical features, imaging characteristics, recurrence risks, and treatment strategies of these tumors, this review aims to enhance early recognition and informed decision-making among clinicians caring for pediatric patients with hand masses.

## Introduction and background

Tumors of the pediatric hand represent a rare and diverse subset of musculoskeletal neoplasms, encompassing both benign and malignant lesions of bone and soft tissue. Due to the hand’s complex anatomy and critical role in growth and function, even benign tumors can result in significant clinical consequences, including deformity, pain, and impaired development. While most pediatric hand masses are benign, malignant bone tumors, although exceedingly rare, pose a high risk of functional loss and metastatic spread if not promptly identified and treated. Diagnostic delays are common, often due to overlapping features with inflammatory or traumatic conditions, and compounded by the limited representation of pediatric hand tumors in current orthopedic literature. This review aims to provide a comprehensive overview of the most clinically relevant bony and malignant tumors of the pediatric hand, outlining their epidemiology, clinical presentation, imaging characteristics, histopathology, and treatment strategies to support early recognition and informed management in pediatric practice.

## Review

Bone masses 

Enchondromas 

An enchondroma is the most common benign tumor of the hand among the adult population but is relatively rare among the pediatric population, accounting for less than 2% of benign bone tumors [1,2]. Enchondromas equally affect individuals of any gender or race and may arise at any age but are more frequently found during adolescence or early adulthood between the ages of 10 and 40 [3]. Although enchondromas are often left untreated due to their perceived mildness, they can progressively worsen as the child matures, leading to the development of severe finger deformities and the formation of sizable tumor masses [4].

Enchondromas originate from cartilage and typically form within the medullary space of tubular bones, presenting as mature hyaline cartilaginous islands that persist after the completion of endochondral ossification. In the hand, enchondromas may present as solitary lesions or multiple enchondromatoses [5]. Multiple tumors with potential for relapse may also be observed in certain conditions, such as Ollier disease or Maffucci syndrome, which presents with additional soft tissue hemangiomas at an increased risk of malignant transformation [6]. 

Enchondromas are often discovered incidentally on X-rays taken for unrelated reasons, with pain or pathologic fractures being the most commonly reported findings. Radiographs and computed tomography (CT) imaging reveal localized osteolytic lesions with calcification and scalloping of the cortical bone [5]. Magnetic resonance imaging (MRI) shows intermediate signal intensity on T1-weighted images and high signal intensity on T2-weighted images for cartilaginous areas. Histological examination is the definitive diagnostic method for enchondromas, although asymptomatic cases may not require it. 

The treatment of enchondromas depends on the specific circumstances. Small, asymptomatic tumors that show no significant changes in bone tissue or tumor size usually require no surgical treatment and can be managed with regular radiological follow-up examinations [7-9]. Surgical intervention is indicated in cases of pain or pathological fractures. In children, surgical treatment using curettage and corticoplasty without bone grafting has shown satisfactory outcomes by Klein et al [4]. However, care must be taken to avoid damaging the growth plate during curettage, as it can affect growth. When a pathological fracture is also present, the process of curettage is typically postponed until the fracture has healed [10]. However, if the fracture is highly unstable, curettage may be performed alongside defect filling and/or fixation procedures. 

The recurrence rate in enchondroma cases is generally low and occurs gradually, depending on the thoroughness of tissue removal during the initial surgery [11]. It is important to note that while most enchondromas are benign, there is a small risk of transformation into chondrosarcoma, which is characterized by the development of rapidly progressive symptoms such as increasing pain, swelling, or rapid changes in the appearance or size of the tumor. Regular monitoring is crucial for the early detection of malignant transformation. 

Osteochondromas

The most common benign childhood bone tumor is osteochondroma, which accounts for approximately 20-50% of benign bone tumors and 10-15% of all bone tumors [12,13]. Osteochondromas occur most frequently in the first two decades of life and exhibit a mild male predominance (male-to-female ratio of about 1.5-2:1) [13]. Approximately 85% are solitary osteochondromas, and the remainder of ~15% present as more than one lesion in association with hereditary multiple exostoses (HME), an autosomal dominant disorder caused by mutations in the EXT1 or EXT2 tumor suppressor genes [12,14]. HME has an incidence of around one in 50,000 individuals and typically presents in early childhood with a male-to-female ratio of around 3:1 and bilaterality with several osteochondromas developing along the appendicular skeleton [15]. Osteochondromas most commonly present at the metaphysis of long bones (especially the knee region), and solitary osteochondromas are not very rare within the bones of the hand [14-16]. Conversely, HME patients usually develop osteochondromas within the forearm and hand bones (phalanges, metacarpals, and/or carpals), resulting in digital deformity or functional loss in some cases [14,15].

Clinically, osteochondromas often present as a painless, slowly growing mass and are frequently discovered incidentally; symptoms, when they occur, usually result from mechanical irritation or compression of adjacent structures [12,15]. Pain or functional disturbances can arise from complications such as tendon or muscle impingement, nerve or vascular compression, overlying bursitis, or a fracture through the stalk of a pedunculated lesion [15]. Plain radiographs typically suffice for diagnosis, revealing a sessile or pedunculated bony exostosis with cortical and medullary continuity with the host bone, capped by a layer of cartilage (which is radiolucent and often not directly visible on X-ray) [15,17]. Advanced imaging is reserved for specific indications: MRI (or occasionally ultrasound) is used to assess the cartilage cap thickness and evaluate for malignant transformation when suspected, and CT can help delineate the lesion’s osseous anatomy for surgical planning or when the lesion arises in unusual locations [16,17]. Histologically, an osteochondroma consists of a benign hyaline cartilage cap that resembles growth plate cartilage, beneath which endochondral ossification produces trabecular bone continuous with the underlying cortex and marrow of the native bone [15].

Management depends on symptomatology. Asymptomatic osteochondromas are usually managed with observation and periodic follow-up, especially in children and adolescents, whereas surgical excision is indicated for lesions that cause pain, functional impairment, growth deformity, or neurovascular compromise. In the hand, surgical intervention may be warranted for lesions that restrict tendon gliding, joint motion, or cause angular deformities of a digit [17]. Excision should include the entire lesion along with its cartilage cap and perichondrium to minimize the risk of recurrence [17]. Complete resection is typically curative; recurrence is uncommon (on the order of only a few percent) if the osteochondroma is entirely removed, though incomplete excision can lead to regrowth of the cartilage cap and lesion [17]. Malignant transformation into a peripheral chondrosarcoma is the most serious potential complication. This occurs rarely in solitary osteochondromas (<1% of cases) but is more frequent in HME, with an estimated 5-10% of HME patients developing secondary chondrosarcoma over their lifetime [16]. Suspicion for malignant change is raised by new-onset pain, rapid growth of the lesion after skeletal maturity, or worrisome imaging features-for example, a cartilage cap thickness greater than about 2 cm in an adult (or >3 cm in a child), irregular or scattered calcifications in the cap, lytic areas within the lesion, or erosion of adjacent bone [15,18]. Early identification of these signs is critical, as malignant transformation requires prompt evaluation and definitive management (typically wide surgical excision of the chondrosarcoma) [16,19].

Osteosarcomas

Osteosarcoma is the most common primary malignant tumor of bone, which typically is found in the metaphyses of long bones of children and adolescents undergoing rapid bone growth and development [20]. It is a rare diagnosis, with an incidence of 5.6 per one million people in patients under 15 years of age [21-23]. Males are more commonly affected (1.6:1), and taller individuals have a higher risk [12]. Published series indicate that less than 0.4% of all osteosarcomas occur in the hand, and the English literature reports fewer than 60 cases of hand osteosarcoma [24-27]. 

Osteosarcoma has two peaks of incidence, with 75% of patients developing primary osteosarcoma under the age of 20, while the remainder develop osteosarcoma later on as a secondary tumor associated with preexisting bone disorders such as Paget disease or irradiation [28,29]. The presence of preexisting bone lesions and certain genetic mutations, such as those found in the retinoblastoma (RB1) gene, which affects about two-thirds of patients, increases the likelihood of developing osteosarcoma. 

Osteosarcoma manifests as progressively worsening regional pain and swelling, accompanied by a mixed lytic and blastic bone lesion with indistinct infiltrating margins visible on radiographs [12]. The presence of a Codman’s triangle shadow on X-ray is a distinctive feature of osteosarcoma [12,20]. In some cases, osteosarcoma remains asymptomatic until a pathologic fracture occurs, revealing the underlying malignancy [21]. Elevated levels of serum alkaline phosphatase (in approximately 40% of cases) and lactate dehydrogenase (in approximately 30% of cases) may be observed in laboratory tests [22]. The diagnosis of osteosarcoma is confirmed through histological examination, revealing the presence of malignant osteoblastic cells producing woven bone that is focally calcified [23]. These neoplastic cells appear as polygonal and spindled osteoblastic cells of various sizes and stain positive for alkaline phosphatase, osteocalcin, and osteonectin [20]. 

Osteosarcoma in the hand tends to have higher overall survival rates compared to other osteosarcomas [24]. Standard treatment involves a combination of preoperative chemotherapy accompanied by limb salvage surgery, resulting in 60-80% of patients in remission after five years [20,28]. Due to the possibility of metastasis, detecting and resecting any pulmonary metastases greatly contributes to patient survival [21]. Monitoring serum alkaline phosphatase levels following surgery can help track tumor recurrence or metastasis, as fluctuations in its levels may indicate the return of the tumor [30-32].

Osteoid Osteomas 

An osteoid osteoma is a common benign tumor that predominantly affects children and young adults, with a male predominance (3:1 [33,34]. It is usually seen below the age of 25 and rarely found over the age of 40 [33]. Approximately 5-15% of all osteoid osteomas occur in the hand, particularly at the proximal phalanx, carpus, and distal radius [35-37]. The main symptom is a dull, constant pain, especially at rest and during nighttime, which is characteristically relieved by aspirin or nonsteroidal anti-inflammatory drugs (NSAIDs). The pain is attributed to the tumor's secretion of prostaglandin E2 and prostacyclin. Radiographs typically show a central radiolucent nidus surrounded by reactive sclerosis [36]. The diagnosis can be challenging due to other differential diagnoses, but characteristic features include localized swelling, tenderness, erythema, and sclerosis. CT imaging is superior to MR imaging in diagnosing osteoid osteomas, but MR imaging may prove useful in complex cases. Conservative management can be considered for asymptomatic or pain-controlled lesions. Percutaneous radiofrequency thermal ablation is the current treatment of choice for symptomatic osteoid osteomas, with success rates up to 98% [38]. Possible damage to soft tissue structures surrounding the target site resulting from high temperatures, and probe positioning may be a contraindication for the use of this approach [39]. Surgical excision of the nidus, such as via en bloc resection or burr-down with curettage, may be necessary in cases where RFA is not feasible, especially in pediatric patients, to protect nearby neurovascular structures [40]. Recurrence rates are low with the burr-down technique, and grafts are usually not required. Prompt diagnosis and appropriate treatment are crucial to alleviate symptoms and prevent long-term complications associated with osteoid osteoma. 

Giant Cell Tumor of Bone 

Giant cell tumor (GCT) of bone is a locally aggressive, benign bone tumor that is relatively uncommon among the pediatric population and is instead more often seen in young adults [41]. While GCT most commonly occurs at the ends of long bones, it is relatively rare in the hand, accounting for only 2-5% of cases [42]. The metacarpals and phalanges are the most commonly affected hand bones, with the distal radius being the third most common site for GCT in the body [3]. Patients with GCT of bone typically present with pain, swelling, or pathological fractures. Diagnosis involves imaging studies such as radiographs, MRI, and chest CT to assess the extent of the tumor and identify possible lung metastasis. Radiographs reveal lytic lesions surrounded by a thinned cortex. A radiographic classification system categorizes GCT into three grades based on the extent of cortical involvement and soft tissue extension, from Grade I having well-defined margins and latency to Grade III being aggressive with indistinct borders [43]. Treatment strategies for GCT include curettage and bone grafting or cementation for Grade I and II tumors, while Grade III tumors are better managed with en bloc excision and reconstruction. Recurrence rates are higher with curettage, emphasizing the importance of en bloc excision for aggressive lesions [42,44,45]. Reconstruction of the wrist after en bloc excision presents challenges due to the complex anatomy of the wrist joint. Different reconstruction options include osteoarticular grafts, wrist arthrodesis, and various types of bone grafts [46]. The choice of treatment depends on factors such as the patient's functional expectations and the availability of grafts. While amputation is rarely warranted, it may be considered in cases of recurrent or malignant tumors. GCT of the hand has a higher risk of local recurrence and a greater propensity to metastasize to the lungs compared to GCT in other parts of the skeleton [47]. Therefore, caution must be exercised in its treatment, and regular follow-up and surveillance are warranted. 

Carpal Bosses 

A carpal boss, also known as a carpometacarpal boss, manifests as a bony protuberance of the dorsal carpometacarpal (CMC) joints, appearing as a firm lump on the back of the hand [48]. Although the exact cause is often unknown, it is thought to be associated with progressive degenerative wear resulting from repetitive activities [49]. While carpal bosses are more frequently observed in adults, particularly those in their fourth decade of life, they can occasionally present in children as well. The symptoms of a carpal boss can vary, with most cases being pain-free and simply presenting as a noticeable lump. However, some individuals may experience mild pain or a "snapping" sensation in the overlying tendon. To accurately diagnose and develop an appropriate treatment plan, it is recommended to obtain radiographs, which typically reveal degenerative osteophyte formation and the presence of an accessory ossicle [50]. In cases where the carpal boss is pain-free, observation is generally the preferred approach. However, depending on the severity and impact on the individual's quality of life, alternative options such as icing, NSAIDs, or steroid injections may be considered. Surgical excision of the carpal boss has been demonstrated to be effective without reported complications and can provide relief from symptoms if conservative treatments have proven insufficient [48,50]. 

Malignant masses 

Squamous Cell Carcinomas

Cutaneous squamous cell carcinoma (SCC) is the most common primary malignancy of the hand, with the majority located on the dorsum of the hand and a rare occurrence on the palm [51-54]. Risk factors associated with tumor development in this area include exposure to carcinogens, congenital conditions, suppressed immunity, bacterial or viral infections, local radiation exposure, and trauma to the affected digit [55]. Diagnosis is based on histologic criteria, but distinguishing SCC from reactive or inflammatory processes like chronic dermatitis, epidermoid cysts, and keratoderma, among others, can be challenging, leading to delays in diagnosis, particularly in pediatric patients [55]. Treatment strategies for cutaneous SCC involve surgical excision and take into consideration recurrence rates, preservation of hand function, patient expectations, and potential adverse reactions [56, 57]. Mohs micrographic surgery is recommended for high-risk cases, while standard excision with margin assessment is suitable for low-risk primary tumors [49]. Amputation is controversial but has been associated with higher death rates from metastasis compared to radiotherapy alone [55]. In the case of pediatric nail unit SCC, maintaining full digit utilization is crucial, and treatment options include local and wide excision, Mohs surgery, amputation, and topical chemotherapy [58]. Mohs surgery has proven most effective in preventing recurrence and preserving function. Overall, while SCC of the hand is challenging to diagnose and treat, appropriate management strategies can lead to favorable outcomes in pediatric patients. 

Fibrosarcomas

Fibrosarcoma of the hand is an extremely rare soft-tissue tumor that primarily affects children between birth and 15 years of age [59]. It is most commonly found in the extremities, with the upper extremities being more frequently affected. Unlike fibrosarcoma in adults, congenital fibrosarcoma has a low metastasis rate and a high long-term survival rate. Infantile fibrosarcoma, a subtype of fibrosarcoma, usually presents in the first five years of life and is characterized by a high local recurrence rate but a low metastatic potential [59]. While the exact cause of infantile fibrosarcoma is still unknown, gene fusions due to translocation and certain trisomies have been reported [60]. On histological examination, infantile fibrosarcoma is characterized by spindle cells in interweaving bundles and a myxoid background [61]. 

The clinical presentation of infantile fibrosarcoma includes a local, progressive mass in the distal parts of the extremities [62]. The appearance of the lesion may resemble vascular malformations such as hemangiomas, leading to misdiagnosis [54]. Magnetic resonance imaging (MRI) is a valuable tool for the evaluation and follow-up of soft tissue masses, providing better diagnostic information [63]. MRI findings of infantile fibrosarcoma typically show a heterogeneous mass with a mixture of solid and cystic regions, along with heterogeneous enhancement. Angiography may reveal hypervascularity with disorganized vascular channels and tortuous draining veins [61]. The mainstay of treatment for infantile fibrosarcoma is wide surgical excision, with efforts made to spare the limb whenever possible [59]. However, due to the aggressive nature of the disease and the challenges in achieving clear margins while preserving vital structures, complete resection can be difficult. In such cases, neoadjuvant chemotherapy may be employed to reduce the size of the tumor prior to surgery. The most effective chemotherapy regimen for infantile fibrosarcoma includes vincristine, actinomycin D, and cyclophosphamide [64]. Studies have shown that a significant percentage of patients with infantile fibrosarcoma respond well to this chemotherapy regimen, resulting in tumor reduction. In fact, Orbach et al reported a 71% response rate to vincristine-actinomycin D chemotherapy, with a 5-year survival rate of 89% in these patients [65]. Chemotherapy is particularly recommended for older children to decrease the likelihood of metastasis. Distant metastasis is uncommon, and the prognosis for infantile fibrosarcoma is generally favorable, with high five-year survival rates reported [66]. 

*Melanoma*s 

Pediatric melanoma is a rare form of melanoma in children, accounting for only 0.7% of all melanoma diagnoses in the pediatric population according to Lange et al [67,68]. While cutaneous melanoma is most commonly found in Caucasian populations, melanoma of the hand, including subungual melanoma, affects non-white populations as frequently or even more often [69]. Acral lentiginous melanoma is the most common subtype found in darkly pigmented patients [70]. Congenital and infantile melanoma, although rare, can occur as de novo lesions or as transplacental metastasis from a primary melanoma in the mother [71]. Approximately 50% of childhood melanomas are associated with pre-existing lesions, most commonly giant congenital melanocytic nevi [72]. These melanomas tend to develop in the dermis, have a higher risk of metastasis, and carry a poor prognosis. 

Diagnosing pediatric melanoma requires careful evaluation and consideration of clinical features. The ABCDE system, which stands for asymmetry, border irregularity, color variation, diameter greater than 6 mm, and evolution over time, can be useful in visually assessing pigmented lesions and determining their propensity for malignancy [73]. Dermoscopy, a method of evaluating skin lesions with a magnifying lens, and in vivo confocal laser scanning microscopy can aid in diagnosis [74]. A full-thickness biopsy is necessary for a definitive tissue diagnosis, and histologic evaluation involves various stains and the identification of architectural features. MRI may be considered to assess deeper invasion and proximity to underlying structures [75]. Surgical excision is the primary treatment approach for pediatric melanoma, although recommendations are often extrapolated from adult guidelines [76]. The hand, especially the webspaces and skin over the proximal phalanges, is considered a "danger zone" for metastasis due to the presence of the superficial digital lymphatic plexus [77]. As a result, in the context of pediatric melanoma, sentinel lymph node biopsy (SLNB) is particularly useful [78-80]. Studies have shown that pediatric patients with melanoma have a higher incidence of SLN involvement compared to adults, which indicates a greater likelihood of cancer spread [81-84]. Detecting lymph node involvement through SLNB can help guide further treatment decisions, such as the need for lymph node dissection or adjuvant therapy. High-dose interferon and IL-2 have been studied in pediatric patients, but their efficacy is still being determined [83,84]. Vemurafenib and ipilimumab are two newer FDA-approved agents for metastatic melanoma in adults that have yet to be approved for pediatric use [83,84]. 

*Rhabdomyosarcomas* 

Pediatric rhabdomyosarcoma (RMS) of the hand is a rare but aggressive tumor primarily affecting children. RMS accounts for over 50% of soft tissue sarcomas in children, with an annual incidence of around four cases per million individuals under the age of 20 [85]. This tumor has a tendency to invade nearby structures and can metastasize through the lymphatics and bloodstream. Unlike other soft tissue sarcomas, RMS is generally highly responsive to chemotherapy, resulting in cure rates of approximately 70%. RMS can occur in various locations throughout the body, with the head-neck region and genitourinary tract being the most common sites [86-90]. However, when RMS arises in the extremities, such as the hand, it often presents with the unfavorable alveolar subtype and is associated with older age, a higher frequency of nodal involvement, and a worse prognosis compared to RMS in other locations [91]. 

Rhabdomyosarcomas are malignant neoplasms with striated muscle differentiation [92]. They can be classified into subtypes such as spindle cell, embryonal, botryoid, alveolar, and undifferentiated, with each subtype being more prevalent in specific age groups [93]. The alveolar subtype, accounting for 32% of all rhabdomyosarcomas, exhibits a more aggressive clinical course and poor prognosis. The clinical presentation of rhabdomyosarcoma is often a painless mass with associated symptoms due to mass effect on surrounding structures. Diagnosis requires biopsy and further pathological investigation [92]. Microscopically, the appearance of RMS can resemble other small round blue cell tumors, making immunohistochemical stains such as myogenin and desmin crucial for accurate diagnosis. Cytogenetic analysis can also be performed to distinguish subtypes. Bone invasion is observed in approximately 25% of cases, with bone metastases appearing predominantly as lytic lesions [91]. Imaging techniques, such as ultrasound and MRI, are helpful in evaluating the tumor but lack specific characteristics. 

The treatment strategy for pediatric rhabdomyosarcoma of the hand typically involves a multimodal approach, including surgery, chemotherapy, and radiotherapy [94]. Primary excision is pursued if complete, non-mutilating resection is deemed feasible; otherwise, a biopsy is taken, and chemotherapy and/or radiotherapy are administered to reduce the tumor size before subsequent surgery. Radiotherapy is recommended for patients at risk of local failure, especially after marginal resection or for large tumors. Chemotherapy regimens, such as VAC (vincristine, actinomycin-D, cyclophosphamide), VACA (vincristine, actinomycin-D, cyclophosphamide, adriamycin), IVA (ifosfamide, vincristine, actinomycin-D), or other combinations, have been utilized [95]. Response to chemotherapy is evaluated based on the reduction of tumor size, with complete response, partial response, and stable disease being the main categories. 

The prognosis for pediatric rhabdomyosarcoma of the hand is generally poorer compared to RMS in other anatomical sites, mainly due to a higher incidence of metastatic spread [94,96,97]. The presence of the alveolar subtype and distant metastases further worsens the prognosis. The use of multiple-agent chemotherapy regimens has significantly improved survival rates. Recurrence of RMS can occur, necessitating ongoing monitoring and potential adjustment of the treatment approach. 

**Figure 1:**
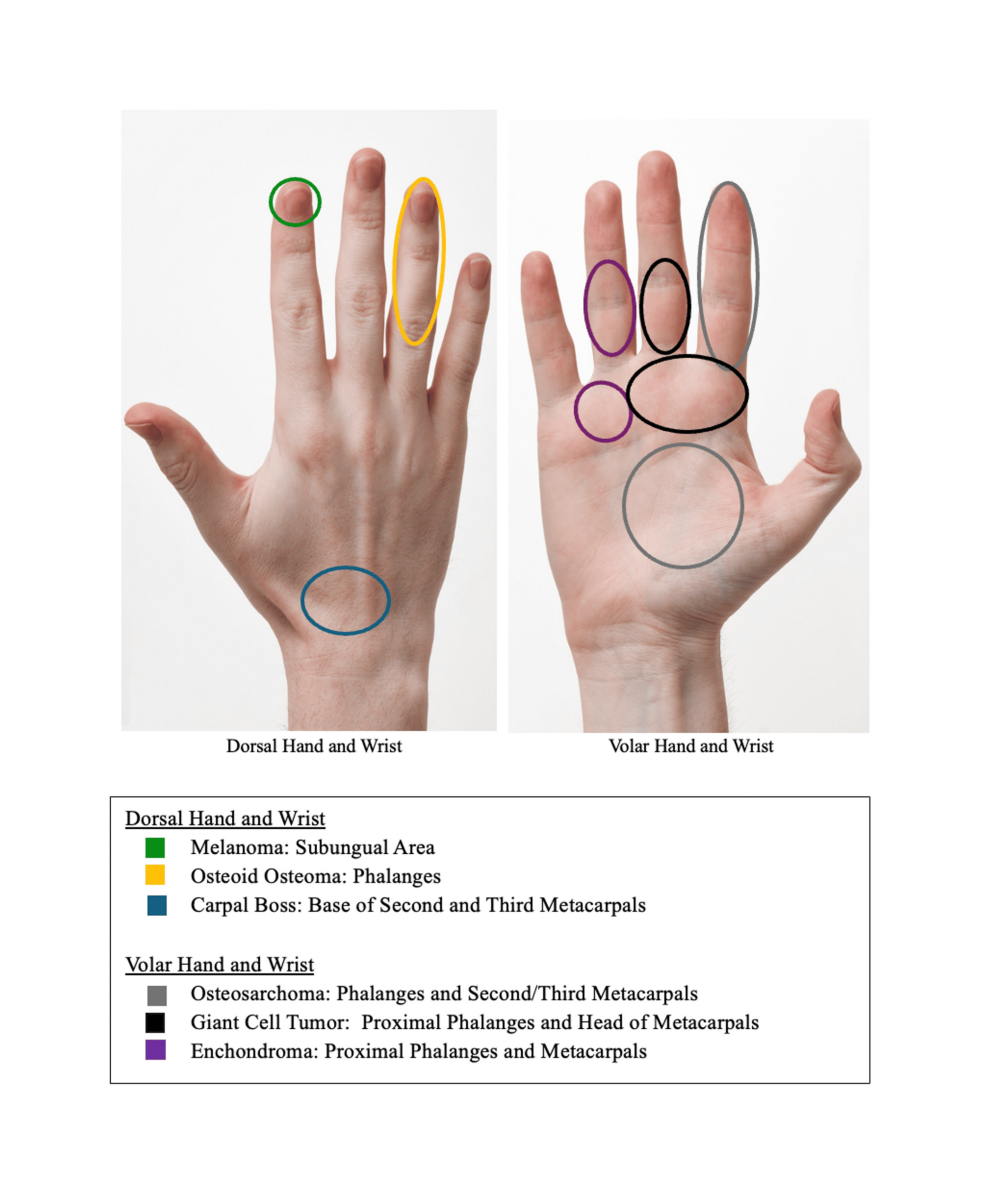
Most common locations of malignant and bony tumors in the pediatric hand. Dorsal view (left) and volar view (right) illustrate typical sites for melanoma (green) [69], osteoid osteoma (yellow) [35-37], and carpal boss (blue) on the dorsal side [48]; and osteosarcoma (gray) [20], giant cell tumor (black) [3], and enchondroma (purple) on the volar side [5]. Not pictured: Osteocarcinomas may involve phalanges, metacarpals, or carpal bones. Fibrosarcomas are common throughout the upper extremities. Hand images sourced from "Human-Hands-Front-Back" by Evan-Amos, licensed under CC BY-SA 3.0 [98].

Figure 1 highlights the most common anatomical locations of certain tumor types discussed within the pediatric hand.

## Conclusions

Pediatric hand tumors, although rare, demand timely recognition and thoughtful management due to their potential for significant functional and oncologic consequences. This review highlights the clinical features, diagnostic approaches, and treatment strategies for the most relevant bony and malignant tumors in this population. A thorough understanding of these lesions can aid clinicians in early diagnosis, minimize unnecessary delays, and guide interventions that preserve both oncologic safety and hand function in growing children.
